# Recognizing states of psychological vulnerability to suicidal behavior: a Bayesian network of artificial intelligence applied to a clinical sample

**DOI:** 10.1186/s12888-020-02535-x

**Published:** 2020-03-30

**Authors:** Jorge Barros, Susana Morales, Arnol García, Orietta Echávarri, Ronit Fischman, Marta Szmulewicz, Claudia Moya, Catalina Núñez, Alemka Tomicic

**Affiliations:** 1 Psychiatry Department, School of Medicine, Pontificia Universidad Católica de Chile, La Reconquista 498, Las Condes, Santiago, Chile; 2 Millennium Institute for Research in Depression and Personality MIDAP, Santiago, Chile; 3 Independent mathematical engineer, Santiago, Chile; 4 School of Nursing, San Sebastian University, Santiago, Chile; 5 School of Psychology, Diego Portales University, Santiago, Chile

**Keywords:** Suicide, Mood disorders, Artificial intelligence, Bayesian models

## Abstract

**Background:**

This study aimed to determine conditional dependence relationships of variables that contribute to psychological vulnerability associated with suicide risk. A Bayesian network (BN) was developed and applied to establish conditional dependence relationships among variables for each individual subject studied. These conditional dependencies represented the different states that patients could experience in relation to suicidal behavior (SB). The clinical sample included 650 mental health patients with mood and anxiety symptomatology.

**Results:**

Mainly indicated that variables within the Bayesian network are part of each patient’s state of psychological vulnerability and have the potential to impact such states and that these variables coexist and are relatively stable over time. These results have enabled us to offer a tool to detect states of psychological vulnerability associated with suicide risk.

**Conclusion:**

If we accept that suicidal behaviors (vulnerability, ideation, and suicidal attempts) exist in constant change and are unstable, we can investigate what individuals experience at specific moments to become better able to intervene in a timely manner to prevent such behaviors. Future testing of the tool developed in this study is needed, not only in specialized mental health environments but also in other environments with high rates of mental illness, such as primary healthcare facilities and educational institutions.

## Background

Decades of research have repeatedly demonstrated that our ability to correctly assess the risk of suicidal behavior (SB) is very limited [9, 16, 38, 39]. A recent systematic review using a meta-analysis of 44 studies conducted between 1945 and 2013 showed a rate of 147/100,000 inpatient suicides, which the authors described as “unacceptable mortality in psychiatric hospitals.” The results suggested that the rate of inpatient suicides may have risen over recent decades [40].

While it is certainly difficult to anticipate suicidal behavior (SB) during hospitalization, it is equally difficult once a patient has been discharged. A meta-analysis of 100 studies of SB and ideation, which were chosen from a total of 11,449 studies originally selected, showed that the greatest risk of suicide occurred during the first 3 months after discharge. Such risk was estimated at a rate of 1132/100,000 during the first 3 months, which slowly declined depending on the follow-up period.

The risk decreased to 494/100,000 when follow-ups were between 1 and 5 years after discharge, to 366/100,000 for follow-ups between 5 and 10 years after discharge, and to 277/100,000 for follow-ups 10 years or more after discharge. The authors noted that studies using data collected after 1995 show much higher suicide rates [9].

Another meta-analysis titled “50 Years of Research on Risk Factors and Suicidal Ideation” [12] concluded that “the ability to predict suicide has not improved in 50 years of research” (p.191). For a better understanding of SB, the authors suggested conducting follow-up studies, where findings should be combined in a complex and replicable way.

Our use of DM and AI tools is intended to complement the use of traditional statistics. This type of analysis adds utility when processing large volumes of data, which requires an understanding of complex phenomena. Such understanding is possible through observing unexpected relationships and conditional dependencies in the data and investigating beyond the hypotheses that may have been established a priori.

Other authors have used MD techniques to study the possible association of clinical and/or demographic variables with past or future suicidal behavior. This approach was used in several very relevant works carried out by different groups [3, 22]. We take a different approach since we want to recognize which configuration of variables is most likely at the time of the risk of SB.

In fact, two studies published in 2017 that used machine learning techniques revealed the advantages of such techniques for studying SB ( [30]; C. G [39].). Overall, the evidence mentioned above has suggested that a paradigm shift is needed [15]. Such a shift can be achieved by studying populations in primary care facilities who are in states that precede SB [16]. Considering the fluctuating nature of SB, efforts to explore the uniqueness of each factor related to its preceding psychological conditions may be challenging.

We recognize the need to consider the simultaneous presence of variables as well as the existence of new factors in SB research. Within the category of “new factors,” we include groups of variables that operate simultaneously. In other words, a new factor might constitute a configuration of variables that emerge at a particular time [5, 23, 25]. While there is still a lack of reliable tools to predict suicide, certain factors that are predominant when a person attempts to end his or her life can be established [36, 42]. Several researchers have studied mental states preceding SB by using different methodologies. Based on information provided by therapists, Hendin, Maltsberger, Haas, Szanto, and Rabinowicz (2004) compared reports of the affective states of patients who attempted suicide to those of control participants. The researchers suggested that “despair” was the most prevalent emotional state in participants who attempted suicide.

Tucker et al. [35] proposed the diagnostic entity “acute suicidal affective disturbance” (ASAD) to describe a common clinical condition among those exhibiting SB that can be deemed independent from their overall diagnoses. ASAD has been validated with the ASADI-L diagnostic instrument, which, according to the researchers, should be prospectively evaluated in individuals as well as through family studies. Galynker et al. [13] recently used the Suicidal Crisis Inventory (SCI) to study SB in a group of inpatients for four to 8 weeks. This scale has 49 items exploring five traditional factors: entrapment, panic-dissociation, ruminative flooding, fear of dying, and emotional pain. Among these factors, SB is mostly plausible based on higher indices for entrapment. Methods that have been tested to assess SB are complementary. Each method can be used to identify risks for subgroups of individuals with SB. For instance, the ASADI-L particularly emphasizes risk assessment in patients with prior suicidal intents, and the SCI evaluates emotional states preceding SB, as shown in the work carried out by Hendin.

We believe that several affective states may interact to place an individual in a state of vulnerability. Considering the complexity of the present task, we favored factor selection by means of a mathematical model. The predictive value of our model should be assessed in future studies by using a strategy similar to that used by Galynker et al. [13]. By focusing on the detailed study of clinical conditions that accompany SB, we also aimed to reveal the variables that can be modified to move patients away from a state characteristic of SB toward a state characteristic of individuals without SB. With this goal in mind, we used various data mining (DM) techniques, namely, support vector machine (SVM), decision tree (DT) and Bayesian network (BN), to specify unique relationships between variables contributing to the state of psychological vulnerability associated with SB.

Over the years, research has identified different risk factors for SB, among which psychiatric disorders, especially depressive disorders [43], and previous suicide attempts [4] have been deemed the most important. Along with depression, other mental disorders, such as schizophrenia, bipolar disorder, substance abuse disorder, eating disorder and borderline personality disorder, are highly present in individuals with suicidal behavior [43]. Likewise, alexithymia has been linked to an increased risk of suicide through the intensification of the propensity to develop depressive symptoms or the enhancement of psychological distress [10]. The literature has described a long list of risk factors in the social, family, biological and personality realms. Nevertheless, the purpose of this study is not to delve into the list of factors (see [43]) but to propose a way in which such risk factors can be analyzed with a novel technique to deepen our understanding of each factor’s dynamic and individual presence.

We used the structure of the data from our first two DM experiments (SVM and DT) to explore the findings that emerged regarding the difference between protective and risk factors [5, 25]. Subsequently, we delved deeper into the dependence relationships among the variables by using the Bayesian network technique [33]. This technique provided a perspective on the conditional dependence relationships among variables in individual cases. The findings of this in-depth study should be used to provide recommendations for clinical interventions.

We acknowledge that suicide risk is highly individual and represents a critical manifestation of particular configurations of common factors. In other words, this study sheds light on the specificity of the subjective experience of psychological suffering by examining unique configurations of variables that differed from those already identified in the literature. Such configurations were deemed to be multidimensional, with each variable representing specific weights and interacting within conditional dependence relationships [19, 41].

### Main purpose

In light of the above, the purpose of this study was to deepen the understanding of the phenomenon of suicide through the study of psychological variables present in a clinical population that had presented suicidal ideation and suicidal behavior. We aimed to determine the relationships between variables and the existence of conditional dependency relationships^1^ beyond the preliminary hypotheses. The study will allow clinicians to have a tool that is applicable to the particularities of each patient in psychotherapeutic interventions that address both the weaknesses and strengths of patients.

The findings are presented in the results section below.

## Method

The preliminary hypothesis on which the study is based suggests that the more dysfunctional clinical variables that are present, the greater the psychological vulnerability to suicidal behavior.

### Participants

The data analyzed here were drawn from responses from a clinical sample of patients with mood and anxiety symptomatology. Sampling was consecutive and purposive based on the availability of participants. Participants were either ambulatory or hospitalized patients from three mental healthcare centers serving three socioeconomic strata (high, medium and low) in Greater Santiago, Chile.

### Inclusion criteria

The study included female and male participants who were available to participate in the study, who were able to distinguish between fantasy and reality, and who were in an emotional and cognitive state that enabled them to answer the assessment questions. Patients consulting for addiction, eating disorders, psychotic disorders or cognitive disorders were not included to control for the diagnosis variable, but it was recognized that these pathologies can be strongly linked to suicide risk [11, 17, 29]. In addition, data from patients who chose not to participate or who later withdrew from the study were not included.

Participants were undergoing treatment as usual (TAU), which in the case of hospitalized patients consisted of crisis intervention with psychological, psychiatric, and occupational therapy approaches. For outpatients, treatment consisted of psychiatric and psychological approaches. This study was a cross-sectional evaluation of specific moments in participants’ timelines.

Psychiatric diagnoses were made in collaboration with the treating teams according to the diagnostic criteria established in the Diagnostic and Statistical Manual of Mental Disorders, 4th Edition (DSM-IV-TR) [1].

### Prior findings

Previous results from the same group of patients were used for the development of Bayesian networks [27]. SVM analysis provided variables that placed patients in either a risk or no risk condition. DT analyses showed possible configurations of combinations that could be located (depending on the route) in a state of risk or no risk. Such prior results were used to select relevant clinical and personality variables that either made individuals less likely to experience the psychological vulnerability associated with suicidal risk or placed them in such a state. These variables included psychological distress resulting in dysfunctionality, a dysfunctional experience, expression of aggression, factors that prevented suicidal behavior, destructive depressive experiences, and satisfaction with family functioning ([24], 2015; Morales, Echávarri, [5, 26, 34]).

The characteristics of the analyses included in this study are detailed below:

- Support vector machine (SVM) model.^2^ This technique was used to define whether a group was either at risk or not at risk by using supervised learning models linked to learning algorithms that analyzed and recognized patterns. The model generated 22 variables that, depending on the circumstances in which they occurred, defined whether a person belonged within a suicide risk configuration [5].
- Decision tree (DT) model.^3^ This technique was used to process and analyze large quantities of explicative variables. Based on the lowest Gini index [7] and given an appropriate and a sufficient number of questions, it was possible to identify four decision trees and a trajectory of psychological variables, which created a state of vulnerability to suicidal behavior [25]. The progression of these analyses is detailed in the Results section below, where we mention our prior work [5, 25].
- Sociodemographic and clinical information. Several descriptive variables were assessed: demographic, social, clinical, diagnostic, reasons for seeking treatment, and a description of the participant’s behavior or suicidal ideation, when applicable.
- In the present study, we further identified conditional relationships among variables using the graphical model technique [2], specifically the Bayesian network technique [33].

A probabilistic graphical model is defined as a collection of graphs representing conditional probabilities between different variables. The Bayesian network is a type of probabilistic graphical model in which a defining graph fulfills certain specific properties (acyclic and directed).

Selected graph theory concepts are defined below:

- Graph: A collection of nodes or vertices as well as a collection of arcs (or edges) in which each arc connects nodes and is visually represented with lines that join nodes.
- Directed graph: A graph where all the arcs are directed; that is, they have a starting node and an ending node and are represented by arrows on the arcs.
- Acyclic graph: A graph is acyclic if it is directed, and there are no sequences of arcs that start at one node and end at the same node. In other words, there is no “route” that leaves from and arrives at the same node.
- Node parent: Node A is considered a parent of node B if there is a directed edge from A to B.
- Node children: Node C is considered a child of node B if there is a directed edge from B to C.

A Bayesian network consists of the following:

- A network structure represented by a directed acyclic graph (DAG) where there is a collection of nodes, in which each node represents a random variable, and each edge represents a dependency relationship or correlation between variables.
- A probability distribution of parameters that can be deconstructed in a local probability distribution based on arcs found in the graph.
- Codes for conditional dependence relationships among the variables in its graph, revealing joint probability distributions expressed as factorizations of local probabilities, in which joint probabilities of all the variables can be calculated as the products of the probabilities of all the variables given their parent values.

### Instruments

We developed a psychological evaluation instrument [5] available both online and offline. The instrument includes 25 questions to be answered on a Likert scale, and the answers are analyzed based on an algorithm defined by the Bayesian network model. The results identify whether a patient is in a state with SB characteristics. Then, each respondent is placed on a continuum of discomfort/well-being and fragility. This tool also considers risk factors and protective factors. The results from Barros et al. [5] identified areas of interest for particular psychotherapeutic interventions for each respondent: a) feelings of satisfaction/dissatisfaction with life; b) state of satisfaction/dissatisfaction with oneself and achievements; and c) reasons to live/stay alive if you are thinking about committing suicide. An example of the results is presented in the results section below.

### Data collection

Potential participants were asked to sign a written informed consent form and were then asked to respond to the questions included in the following instruments: Outcome Questionnaire (OQ-45.2), State-Trait Anger Expression Inventory (STAXI-2), Reasons for Living Scale (RFL), Depressive Experience Questionnaire (DEQ), Family APGAR, and sociodemographic and clinical questionnaires. Detailed descriptions of these questionnaires can be found in Barros et al. [5] and Morales et al. [25].

Participants were guided through the questionnaires and written consent form by specially trained evaluators. If participants were minors, the consent form was signed in writing by their guardian or caregiver. The probability and protocol were approved by the institutional ethics committees of the School of Medicine at the Catholic University of Chile and the Sótero del Río Hospital.

The aims and methodology of the study were explained to participants, as well as the unpaid nature of their participation. Costs, risks of participating in the study, the voluntary nature of participation, the right to withdraw from the study, and confidentiality were also explained. Authorizations from treating physicians were also requested for patients’ participation in the study, and any potential deterioration in the patients’ mental states during the study was to be noted. No incidents were recorded during this study. Participants were also offered the opportunity to inquire further about the study by contacting the principal investigator. Health clinicians, researchers, and mathematical analysts collaborated in offering assistance to participants throughout the study.

### Descriptive analysis of the data

Participants were categorized into the following two groups: 1) with suicidal behavior, as indicated by attendance of consultations regarding a suicide attempt or suicidal ideation within the preceding year (*n* = 326); 2) without suicidal behavior, as indicated by attendance of mental health consultations with no suicide attempt or signs of suicidal ideation within the preceding year (*n* = 324). The sample included 650 ambulatory mental health patients between the ages of 14 and 85 (adolescents, young adults, adults, and seniors) who were recruited between June 2010 and December 2014. Of this sample, 95.38% had been diagnosed with mood disorders (DSM IV-R). Among the total sample, the average age was 39.77 ± 15.03, with an age range between 14 and 83 years old. There were 517 women (79.54%) and 133 men (20.46%). Sociodemographic characteristics are detailed in Table 1.

**Table 1:** Sociodemographic characteristics of the sample, differences between groups

Variable	Total	Group without current suicidal behavior	Group with suicidal behavior	Test
Women	517 (79.54%)	257 (79.32%)	260 (79.75%)	
Men	133 (20.46%)	67 (20.68%)	66 (20.25%)	
Chi-squared Test				X = 0.001583
				df = 1
				*p*-value = 0.9683
Age				
Average	39.77	42.13	37.42	
Standard deviation	15.03	14.8	14.91	
T-Student Test				t = 4.041
				df = 648
				*p*-value = 5.955e-05 **
Education level				
Without higher education	336 (51.69%)	178 (54.94%)	158 (48.47%)	
With higher education	314 (48.31%)	146 (45.06%)	168 (51.53%)	
Chi-squared Test				X = 2.473
				df = 1
				*p*-value = 0.1158
				*p*-value = 0.244
Living with				
Alone	67 (10.31%)	31 (9.57%)	36 (11.04%)	
Couple	84 (12.92%)	49 (15.12%)	35 (10.74%)	
Family	499 (76.77%)	244 (75.31%)	255 (78.22%)	
Chi-squared Test				X = 2.943
				df = 2
				*p*-value = 0.2296
Marital status				
Single	271 (41.69%)	115 (35.49%)	156 (47.85%)	
Married	238 (36.62%)	135 (41.67%)	103 (31.6%)	
Free union	31 (4.77%)	18 (5.56%)	13 (3.99%)	
Separated o widowed	110 (16.92%)	56 (17.28%)	54 (16.56%)	
Chi-squared Test				X = 11.34
				df = 3
				*p*-value = 0.01001 *
Children				
Average	1.438	1.373	1.503	
Standard deviation	1.556	1.509	1.601	
T-Student Test				t = −1.062
				df = 646.2
				*p*-value = 0.2887
Occupation				
Within_working_force	329 (50.62%)	187 (57.72%)	142 (43.56%)	
Student	142 (21.85%)	52 (16.05%)	90 (27.61%)	
Unemployed	39 (6%)	18 (5.56%)	21 (6.44%)	
Housewife	127 (19.54%)	60 (18.52%)	67 (20.55%)	
not_working	13 (2%)	7 (2.16%)	6 (1.84%)	
Chi-squared Test				X = 17.01
				df = 4
				*p*-value = 0.001923 *

Note: *p < 0.05; **p < 0.001

The total sample was also mainly characterized by patients diagnosed with affective disorders, most commonly major depressive disorder (43.38%; *n* = 282), of whom 28.09% (*n* = 91) had not exhibited SB (attempt or ideation) during the past year, and 58.59% (*n* = 191) had attempted suicide. Of the 191 patients with SB who had been diagnosed with major depressive disorder, 26.18% (*n* = 50) made high-severity suicide attempts, 19.90% (*n* = 38) made low-severity suicide attempts, and 53.93% (*n* = 103) presented suicidal ideation. Low-severity suicide attempts were characterized by minimal intentions of dying, the low subjective or objective lethality of the attempt, and the deployment of efforts to be saved after the suicide attempt. On the other hand, high-severity suicide attempts were characterized by strong intentions to die as well as high subjective and objective lethality, with no efforts to be saved being made after the attempt. The psychiatric diagnoses are shown in Table 2.

**Table 2:** Psychiatric diagnoses distribution, differences between groups

Variable N (%)	Total	Group without current suicidal behavior	Group with suicidal behavior		Suicide attempt high severity	Suicide attempt low severity	Suicide ideation	Sub-total
Major depressive disorder	282 (43.38%)	91 (28.09%)	191 (58.59%)		50 (26.18%)	38 (19.90%)	103 (53.93%)	191
Bipolar disorder	105 (16.15%)	59 (18.21%)	46 (14.11%)		8 (17.39%)	12 (26.09%)	26 (56.52%)	46
Moderate depressive disorder	54 (8.31%)	32 (9.88%)	22 (6.75%)		4 (18.18%)	8 (36.36%)	10 (45.45%)	22
Mild depressive disorder	12 (1.85%)	11 (3.4%)	1 (0.31%)		0 (0%)	0 (0%)	1 (100%)	1
Adjustment disorder	70 (10.77%)	45 (13.89%)	25 (7.67%)		5 (20.00%)	12 (48.00%)	8 (32%)	25
Anxiety disorder	75 (11.54%)	53 (16.36%)	22 (6.75%)		4 (18.18%)	4 (18.18%)	14 (63.64%)	22
Mixed episode	15 (2.31%)	13 (4.01%)	2 (0.61%)		1 (50.00%)	0 (0%)	1 (50%)	2
Other disorders	30 (4.62%)	16 (4.94%)	14 (4.29%)		5 (35.71%)	3 (21.43%)	6 (42.86%)	14
Dysthymia	7 (1.08%)	4 (1.23%)	3 (0.92%)		2 (66.67%)	0 (0%)	1 (33.33%)	3
Chi-squared Test				X = 74.12				326
				df = 8				
				*p*-value = 7.397e-13 **				

Regarding the age distribution, the total sample included patients in the following age groups: 14–19 years old (*n* = 76; 11.69%); 20–29 years old (*n* = 119; 18.31%); 30–39 years old (*n* = 123; 18.92%); 40–49 years old (*n* = 125; 19.23%); 50–59 years old (*n* = 146; 22.46%); 60 years and up (*n* = 61; 9.38%). The age distributions are shown in Table 3.

**Table 3:** Age distribution, differences between groups

Variable N (%)	Total	Group without current suicidal behavior	Group with suicidal behavior	Test
14–19 years	76 (11.69%)	24 (7.41%)	52 (15.95%)	
20–29 years	119 (18.31%)	54 (16.67%)	65 (19.94%)	
30–39 years	123 (18.92%)	57 (17.59%)	66 (20.25%)	
40–49 years	125 (19.23%)	72 (22.22%)	53 (16.26%)	
50–59 years	146 (22.46%)	75 (23.15%)	71 (21.78%)	
60 years and more	61 (9.38%)	42 (12.96%)	19 (5.83%)	
Chi-squared Test				X = 23.65
				df = 5
				*p*-value = 0.0002529 **

Note: *p < 0.05; **p < 0.001

### Data analysis

Given the large number of variables currently available, it was necessary to perform a feature selection to narrow down what was to be modeled. This approach follows the principle of parsimony, indicating that if two models show the same performance, the model that has a smaller number of variables would be preferred. Consequently, considering previous work, we based the analysis of this study on two primary explorations using SVM and DT, as explained above. The software was R Project for Statistical Computer (R [31]).

### Initial data processing

The results from the previous SVM analysis provided a model that selected 22 variables, which, depending on the circumstances, could define whether a person was in a suicide risk zone (accuracy = 0.78, sensitivity = 0.77, and specificity = 0.79). The assessment of all these variables allowed a determination of whether a patient was at risk of attempting suicide or was actively thinking of attempting suicide. Interrelationships between these variables were multiple and contributed to the particular ways in which variables were configured for each case. The metrics and analysis are presented in [5].

The results from the DT analysis showed the flow of responses as a trajectory of psychological variables that constituted a current situation of suicide risk (or no risk). Four trees distinguishing the groups were established, and the elements of one tree were analyzed in greater detail since they included both clinical and personality variables. This tree consisted of six nodes without suicide risk and eight nodes with suicide risk. Decision tree 01 had a 0.674 accuracy value, a 0.652 precision value, a 0.678 recall value, a 0.670 specificity value, an F measure of 0.665, and a 73.35% receiver operating characteristic (ROC) area under the curve (AUC). Decision tree 02 had a 0.669 accuracy, a 0.642 precision, a 0.694 recall, a 0.647 specificity, a 0.667 F measure, and a 68.91% ROC AUC. Decision tree 03 yielded a 0.681 accuracy value, a 0.675 precision value, a 0.638 recall value, a 0.721 specificity value, a 0.656 F measure, and a 65.86% ROC AUC. Decision tree 04 showed a 0.714 accuracy value, a 0.734 precision value, a 0.628 recall value, a 0.792 specificity value, a 0.677 F measure, and a 58.85% ROC AUC. The metrics and analysis are described in [25].

**Fig. 1:**
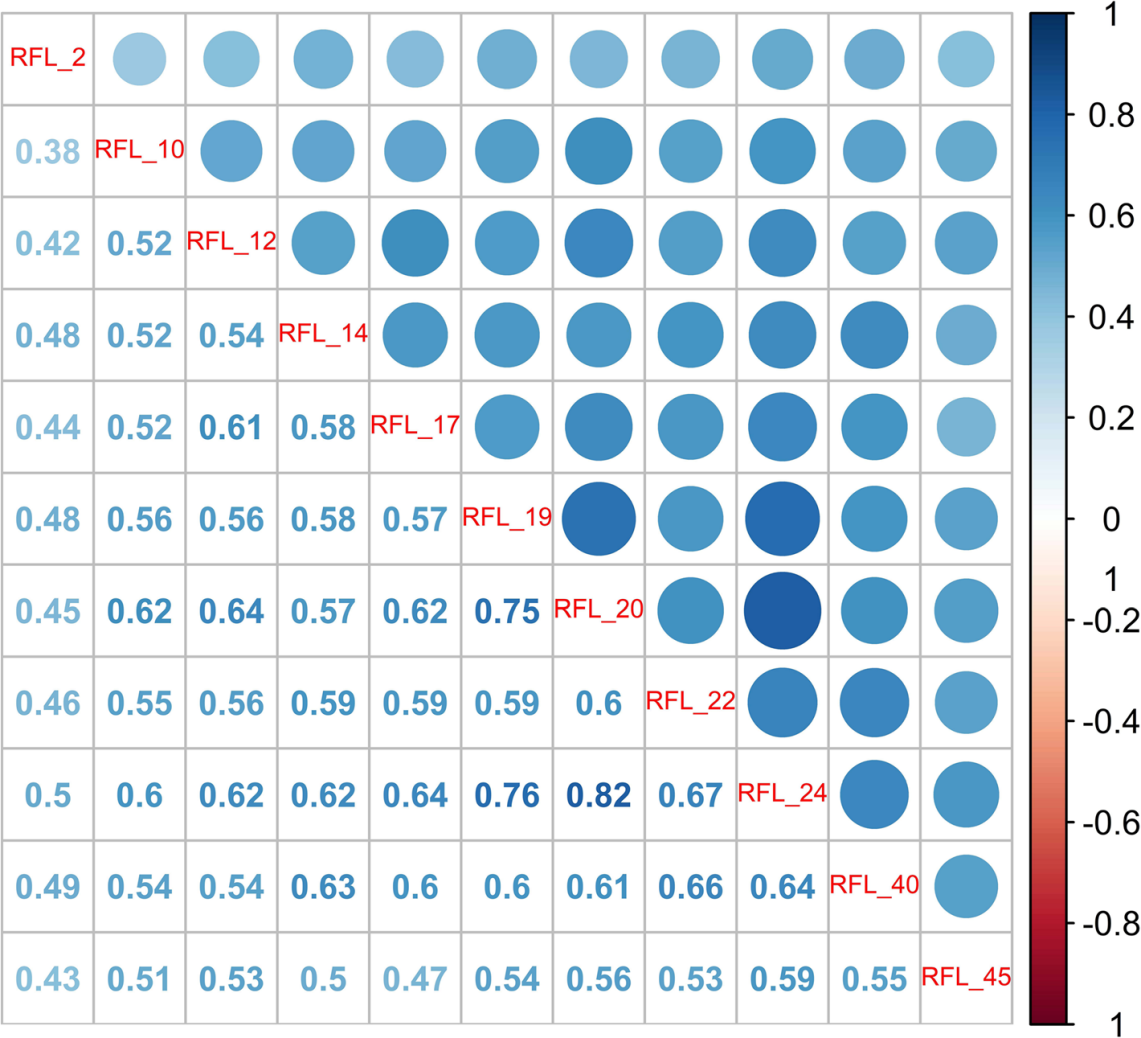
The shows a matrix of correlations between the selected questions of the Reasons for Living Instrument. All the questions presented a high correlation. Query 25 is not in the correlation analysis because its importance suggests its use individually and not combined with the rest of the inquiries

**Fig. 2:**
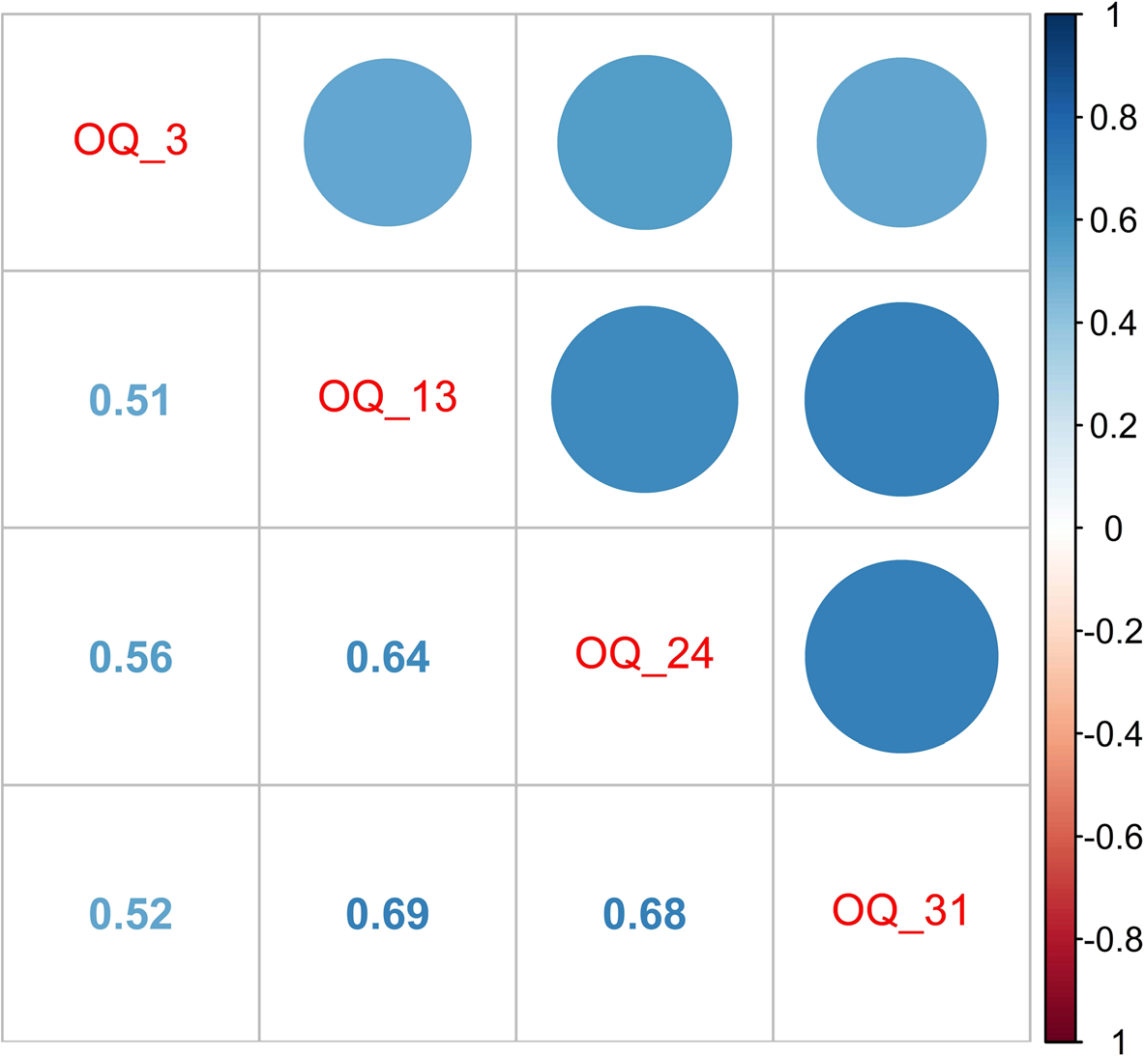
The figure presents a matrix of correlations between the selected questions of the Outcome Questionnaire. The four chosen variables where highly correlated. Query 8 is not in the correlation analysis because its importance suggests its use individually and not combined with the rest of the inquiries

Taking the prior findings as inputs (i.e., the support vector machine and decision tree results mentioned above), we started with 25 variables. These variables were grouped and reprocessed into categories as follows: demographics were categorized as discrete values for classification, the Reasons for Living (RFL) questions were grouped into two variables, and question 25 of the Reasons For Living Questionnaire was kept separate because it was shown to be a relevant variable on its own, while the remaining questions were grouped as a single variable (due to strong correlations among them as seen in Fig. 1) by using the averages of their values; items from the Outcome Questionnaire (OQ) were grouped into a single variable (due to strong correlations among them) by using the averages of their values, except for question 8 from the Outcome Questionnaire, which was kept separate because of its relevance as a question on its own [18, 37]. Figure 2 presents a matrix of correlations between the selected questions from the Outcome Questionnaire.

**Fig. 3:**
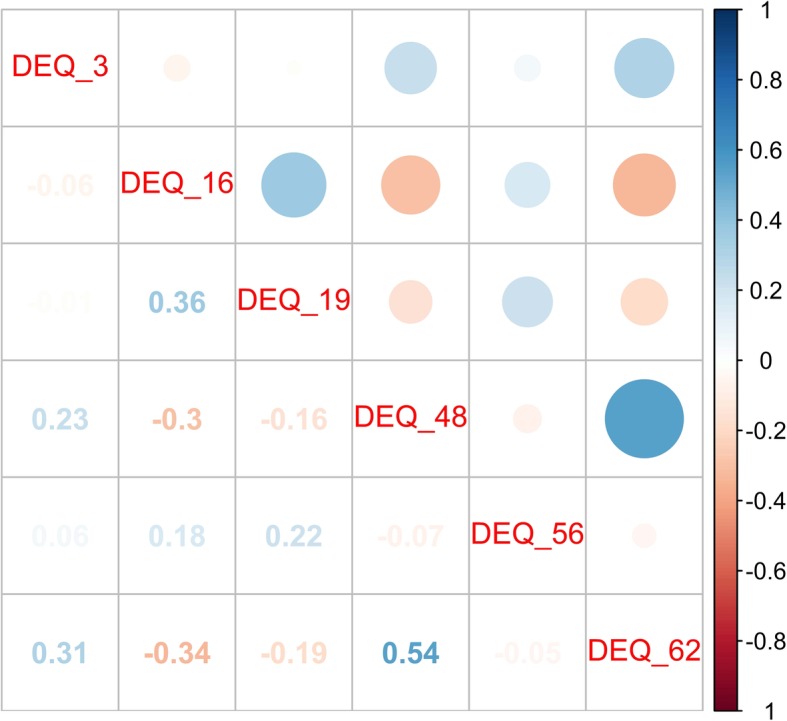
The figure shows a matrix of correlations between the selected questions of the Depressive Experience Questionnaire. The chosen variables do not show a significant correlation between them

The Depressive Experience Questionnaire (DEQ) underwent a different preprocessing procedure. Correlations among the variables were weak (Fig. 3). Therefore, a principal component analysis (PCA) was performed with standardized variables [32], and the first two main components that explained 56.437% of the variance were chosen (Table 4). The first main component, called “low self-esteem”, included the being unable to accept personal plans and goals, having feelings of inner emptiness, becoming terrified when alone, having feelings of personal distress linked to success/failure, being concerned about what others can provide in relationships, and having feelings of dissatisfaction with oneself. This first component had higher coefficients associated with variables DEQ_16 and DEQ_19 and lower scores associated with variables DEQ_56 and DEQ_62, with negative effects on the latter two variables. The second main component, called “interpersonal sensitivity”, included accepting personal plans and goals, setting very high goals, becoming terrified when alone, fluctuating between feeling big and small, not feeling jealous in relationships, and needing things that only others can provide. Variables for the second component all had coefficients greater than 0, and the variables from items 3, 19, and 56 of the DEQ had the greatest impact on this component. The quadrants were configured with the following distribution:

- 00: Patients with low scores for main components 1 and 2 (high self-esteem and low interpersonal sensitivity);
- 01: Patients with a low score for main component 1 and a high score for main component 2 (high self-esteem and high interpersonal sensitivity);
- 10: Patients with a high score for main component 1 and a low score for main component 2 (low self-esteem and low interpersonal sensitivity);
- 11: Patients with high scores for main components 1 and 2 (low self-esteem and high interpersonal sensitivity) (Table 5).

**Table 4:** Ratio of variance explained DEQ Main Components and Coefficients of PC! Also, PC2

	PC1	PC2	PC3	PC4	PC5	PC6
Standard deviation	1.452	1.131	0.905	0.869	0.770	0.668
Proportion of Variance	0.351	0.213	0.137	0.126	0.099	0.074
Cumulative Proportion	0.351	0.564	0.701	0.827	0.926	1.000
Coefficients DEQ_Question_3	−0.277	0.515				
DEQ_Question_16	0.464	0.286				
DEQ_Question_19	0.357	0.471				
DEQ_Question_48	−0.509	0.234				
DEQ_Question_56	0.184	0.555				
DEQ_Question_62	−0.537	0.262

**Table 5:** Coefficients and description of two main components

	PC1	Main Component n° 1	PC1 low self-esteem.
DEQ_PRE_3	−0.277	Disagreeing with the statement “I feel comfortable with my personal plans and goals rather than trying to aim for something more”	(inability to accept personal plans and goals).
DEQ_PRE_16	0.464	Agreeing with the statement “sometimes I feel empty inside”.	(having feelings of inner emptiness).
DEQ_PRE_19	0.357	Agreeing with the statement “I become terrified when I feel alone”.	(becoming terrified when alone).
DEQ_PRE_48	−0.509	Disagreeing with the statement “I feel good within myself whether I succeed or fail”.	(feelings of personal distress linked to success-failure).
DEQ_PRE_56	0.184	Agreeing with the statement “When it comes to my relationships with others, I am very concerned about what they provide me.”	(being concerned with that others can provide in relationships).
DEQ_PRE_62	−0.537	Agreeing with the statement “I am very satisfied with myself and with what I have achieved.”	(feelings of dissatisfaction with oneself and with one’s achievements).
	PC2	Main Component n° 2	PC2 Interpersonal sensitivity.
DEQ_PRE_3	0.515	Agreeing with the statement “in general, I feel comfortable with my personal plans and goals rather than trying to aim for something more”.	(acceptance of personal plans and goals).
DEQ_PRE_16	0.286	Agreeing with the statement “I set myself very high goals”.	(setting very high goals).
DEQ_PRE_19	0.471	Agreeing with the statement “I become terrified when I feel alone.”	(becoming terrified when alone).
DEQ_PRE_48	0.234	Agreeing with the statement “sometimes I feel very big and at other times I feel very small”.	(fluctuating between feeling big and small).
DEQ_PRE_56	0.555	Agreeing with the statement “when I am involved with someone, I never feel jealous”.	(not feeling jealous in relationships).
DEQ_PRE_62	0.262	Agreeing with the statement “I really need things that only other people can give me”.	(needing things that only others can provide).

**Fig. 4:**
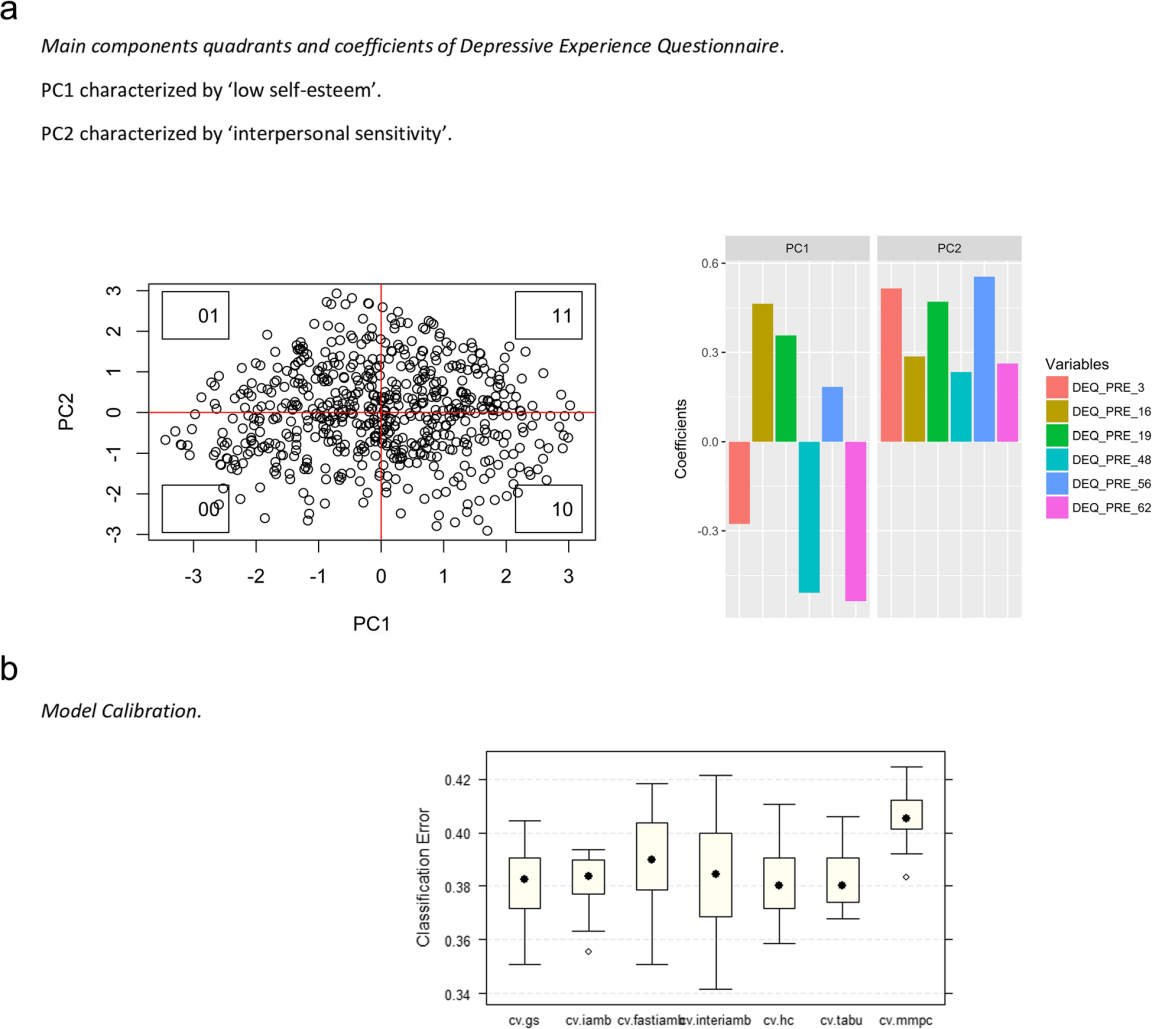
**a** The figure displays a Principal Component Analysis performed with the DEQ variables, selecting the first two principal components. The first graph shows the distribution of the projected points in these two components. The second graph shows the contribution of each of the DEQ questions to the first two principal components. **b** Shows classification error obtained in the model calibration process, using different search algorithms. The Tabu search algorithm was selected due to its lower average classification error and lower variance in the cross-validation sample

The coefficients of the DEQ variables for each of the two components are shown in Table 4 and Fig. 4. Feature transformation for the DEQ variables was necessary to narrow the scope of the problem. Regarding this questionnaire, the selected components rather than the original variables were used to calibrate the Bayesian network model.

With the new feature obtained from the DEQ variables, in the total sample, 24.3% (*n* = 158) of patients had high self-esteem and low interpersonal sensitivity, 24.3% (n = 158) of patients had high self-esteem and high interpersonal sensitivity, 26.3% (*n* = 171) of patients had low self-esteem and low interpersonal sensitivity, and 25,1% (*n* = 163) of patients had low self-esteem and high interpersonal sensitivity.

Meanwhile, participants in the group with SB had the following characteristics: 15.3% (*n* = 50) of patients had high self-esteem and low interpersonal sensitivity, 17.4% (*n* = 57) of patients had high self-esteem and high interpersonal sensitivity, 38.2% (*n* = 125) of patients had low self-esteem and low interpersonal sensitivity, and 29.1% (*n* = 95) of patients had low self-esteem and high interpersonal sensitivity.

### Model calibration

The calibration of the model was achieved using cross-validation. The process consists of two stages:

1. Learning the structure of the network.
2. Learning the parameters of the network.

**Fig. 5:**
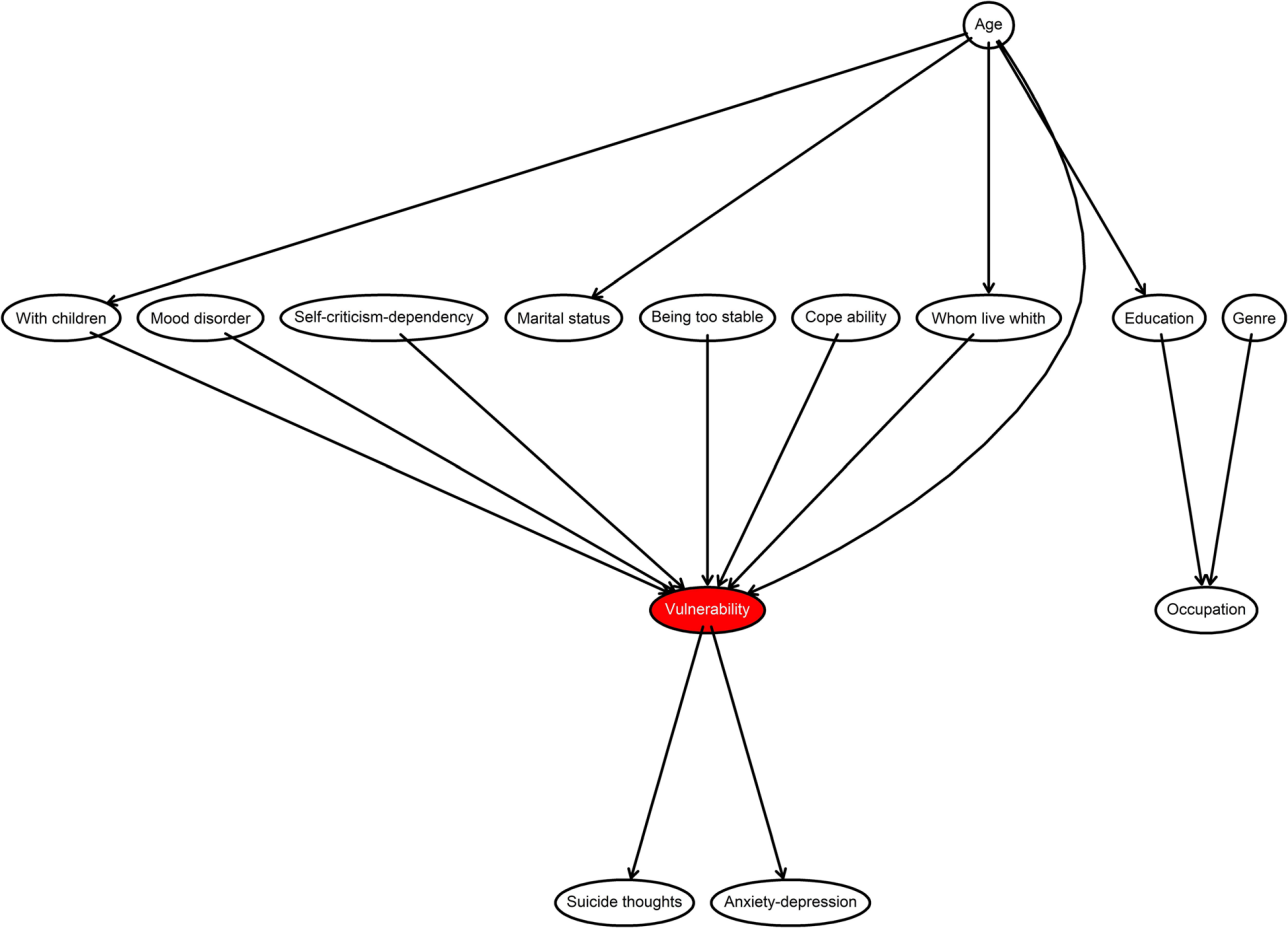
The figure presents the Initial graph with selected clinical and sociodemographic variables based on expert knowledge. The red node represents the response variable associated with suicidal risk

For the first stage, a mixed approach based on a) clinical expertise and knowledge and b) heuristics was used to learn the structure from the data. Based on clinical expertise and domain knowledge, an initial graph was defined regarding the relationships that we wanted to keep in the structure based on their clinical relevance. Additionally, a set of ‘blacklisted’ arcs were defined if the arcs that we did not want were part of the graph. This graph is shown in Fig. 5. Then, the structure of the final graph was completed by using a search algorithm, and several methods were tested in the calibration process (Grow-Shrink, Incremental Association, Fast Incremental Association, Interleaved Incremental Association, Hill-Climbing, Tabu search, and Max-min Parents and Children).

**Fig. 6:**
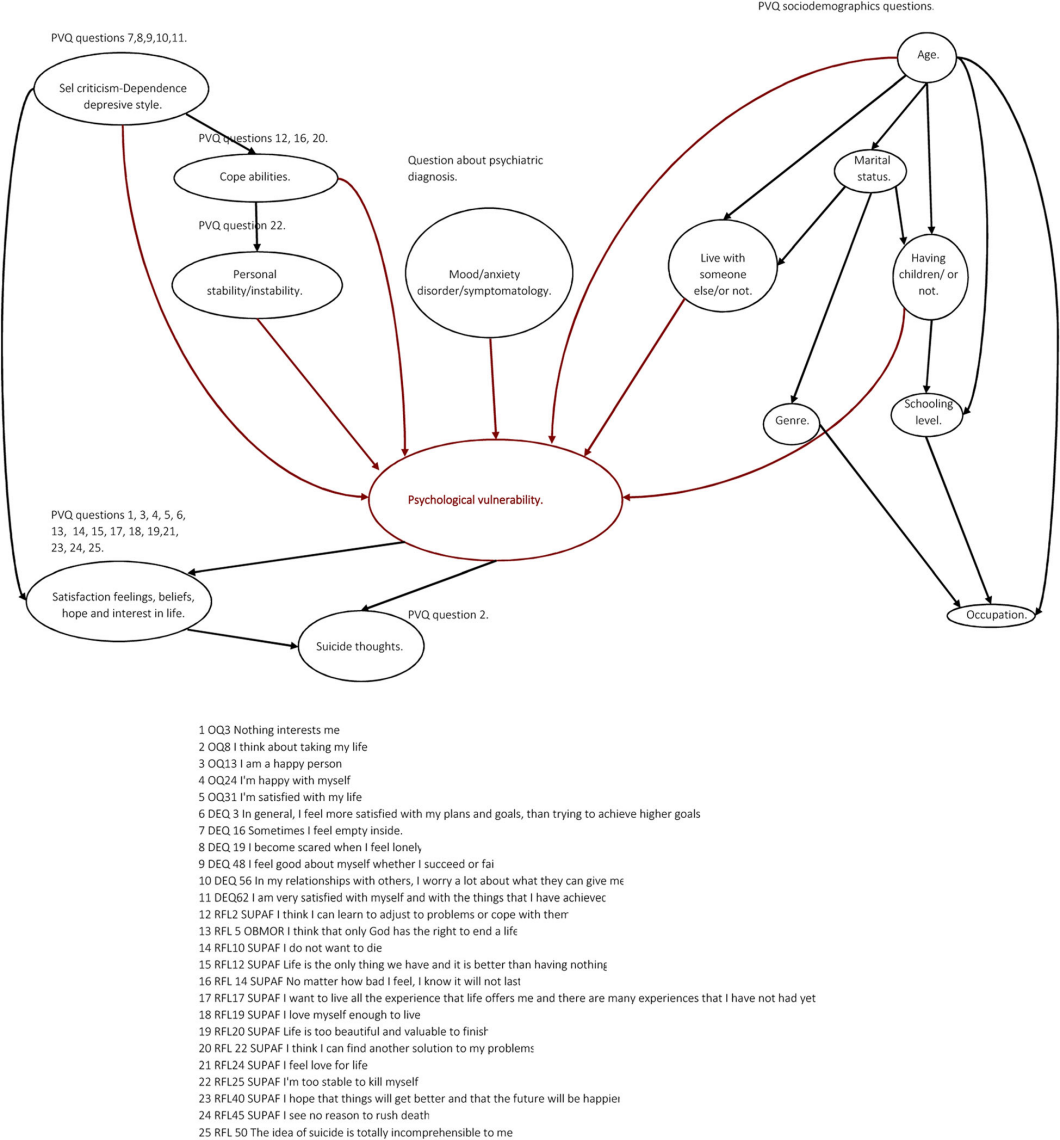
The figure illustrates the final graph after running the TABU algorithm over the complete data and with the initial graph with expert knowledge

New relationships were formed according to the existing correlations in the data, which generated the Bayesian network seen in Fig. 6.

Subsequently, with the structure for each algorithm already in place, parameters associated with joint probability distributions were calibrated using the data and the Bayesian method for estimating parameters. Finally, the tabu search algorithm [28] was selected based on its lower average classification error and lower error classification variance over the cross-validation process. The results of the search algorithm calibration are shown in Fig. 4b.

### Evaluating model fit

As indicated above, the cross-validation technique was used to calibrate the structure and parameters. To determine the model fitness of the calibration process, the same technique was used, and the leave-one-out cross-validation method was also used to evaluate the final model. In both cases, we calculated precision and other relevant performance measures of the resulting model, and the details of each method are presented as follows.

1) The leave-one-out cross-validation method (LOOCV) was used, in which the model was trained with N-1 cases and its accuracy was calculated for the remaining cases (those that were not used for training). This procedure was repeated for each group of data, and the success average was equivalent to the precision estimator. With the LOOCV method, the Bayesian network model fit was 0.7046. The indicators are shown in Table 6.

**Table 6:** Bayesian Network evaluating model fit using Leave-one-out cross-validation (LOOCV)

Metrics	Value
Sensitivity	0.6840
Specificity	0.7253
Pos Pred Value	0.7147
Neg Pred Value	0.6953
Precision	0.7147
Recall	0.6840
F1	0.6991
Prevalence	0.5015
Detection Rate	0.3431
Detection Prevalence	0.4800
Balanced Accuracy	**0.7047**

**Fig. 7:**
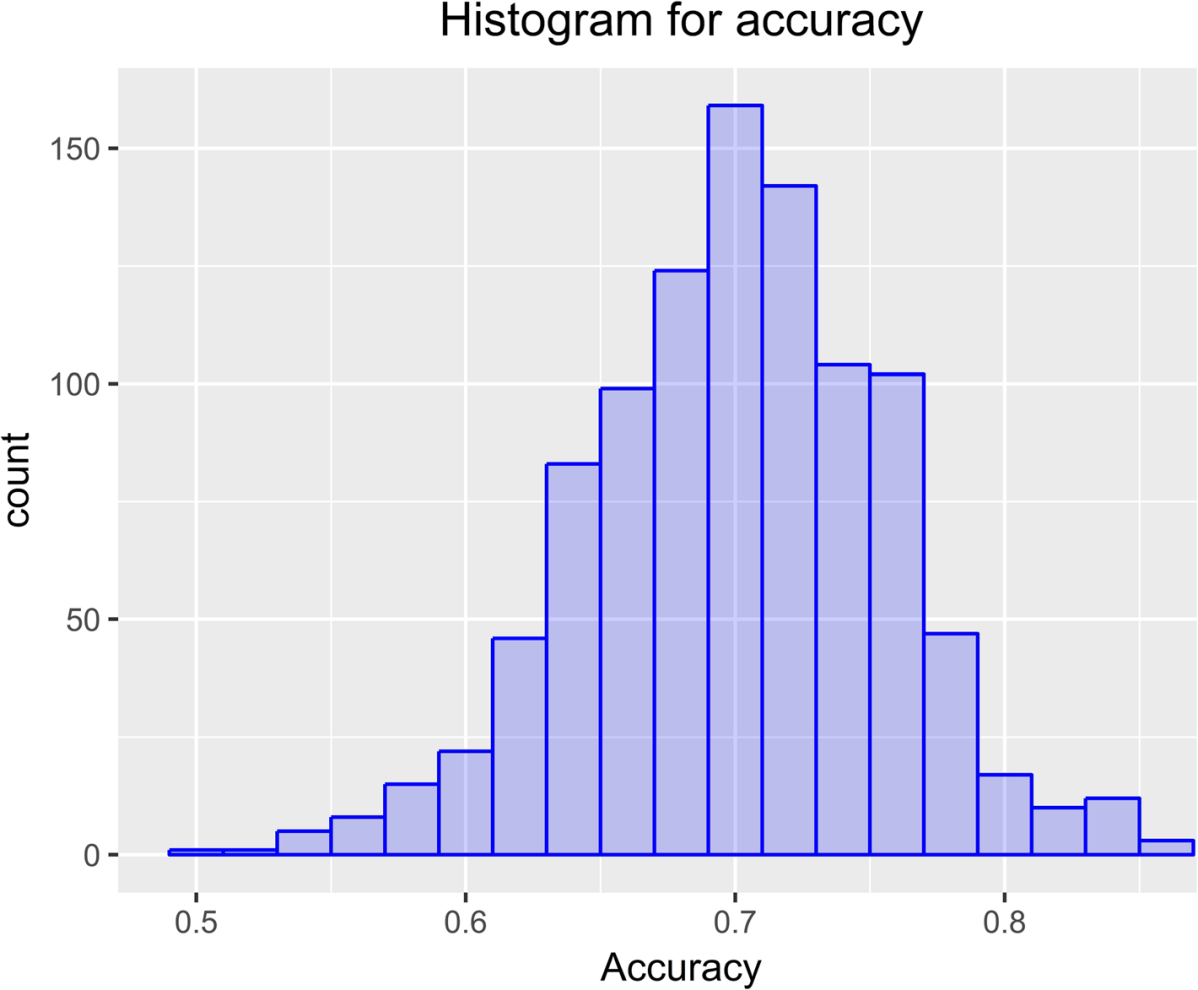
The figure displays model adjustment values distribution obtained in the test sample using cross-validation

2) Repeated 10-fold cross-validation was applied to calculate the model fit by repeating the process 100 times, which gave an average accuracy value of 0.701 (Fig. 7).

The results were used to develop a psychological evaluation questionnaire. This instrument has 25 items that are answered on a Likert scale. These questions can be asked by professionals in contact with individuals who are potentially at risk of attempting suicide. The person administering the questions need not be an expert but should be trained to ask the questions. Details of these questions can be seen in the Psychological Vulnerability Questionnaire shown in Table 7.

**Table 7:** Psychological Vulnerability Questionnaire (PVQ)

		We request to answer the following questions in relation to:							
		How have you been feeling in the last 7 days, including today:	Never	Almost never	Sometimes	Frequently	Almost allways		
1	OQ3	Nothing interests me	0	1	2	3	4		
2	OQ8	I think about taking my life	0	1	2	3	4		
3	OQ13	I am a happy person	4	3	2	1	0		
4	OQ24	I am happy with myself	4	3	2	1	0		
5	OQ31	I am satisfied with my life	4	3	2	1	0		
		**How well does the following phrase describe it?**	**Totally agree 1**	**2**	**3**	**4**	**5**	**6**	**Totally disagree 7**
6	DEQ 3	In general, I feel more satisfied with my plans and goals, than trying to achieve higher goals.	1	2	3	4	5	6	7
7	DEQ 16	Sometimes I feel empty inside.	1	2	3	4	5	6	7
8	DEQ 19	I become scared when I feel lonely	1	2	3	4	5	6	7
9	DEQ 48	I feel good about myself whether I succeed or fail	1	2	3	4	5	6	7
10	DEQ 56	In my relationships with others, I worry a lot about what they can give me	1	2	3	4	5	6	7
11	DEQ62	I am very satisfied with myself and with the things that I have achieved	1	2	3	4	5	6	7
		**Many people have thought about suicide, and others have never considered it. We are interested in the reasons you would have for NOT committing suicide if it ever occurred to you to do so or if someone suggested it to you. How important is each of the following statements to you as a reason NOT to commit suicide?**	**Not important = 1**	**Very unimportant = 2**	**Unimportant = 3**	**Important = 4**	**Very important = 5**	**Extremely important = 6**	
12	RFL2 SUPAF	I think I can learn to adjust to problems or cope with them	1	2	3	4	5	6	
13	RFL 5 OBMOR	I think that only God has the right to end a life	1	2	3	4	5	6	
14	RFL10 SUPAF	I do not want to die	1	2	3	4	5	6	
15	RFL12 SUPAF	Life is the only thing we have, and it is better than having nothing	1	2	3	4	5	6	
16	RFL 14 SUPAF	No matter how bad I feel, I know it will not last	1	2	3	4	5	6	
17	RFL17 SUPAF	I want to live all the experience that life offers me and there are many experiences that I have not had yet.	1	2	3	4	5	6	
18	RFL19 SUPAF	I love myself enough to live	1	2	3	4	5	6	
19	RFL20 SUPAF	Life is too beautiful and valuable to finish	1	2	3	4	5	6	
20	RFL 22 SUPAF	I think I can find another solution to my problems	1	2	3	4	5	6	
21	RFL24 SUPAF	I feel love for life	1	2	3	4	5	6	
22	RFL25 SUPAF	I’m too stable to kill myself	1	2	3	4	5	6	
23	RFL40 SUPAF	I hope that things will get better and that the future will be happier	1	2	3	4	5	6	
24	RFL45 SUPAF	I see no reason to rush death	1	2	3	4	5	6	
25	RFL 50	The idea of suicide is totally incomprehensible to me	1	2	3	4	5	6	
	**Sociodemographics**	Diagnosis	Mild depressive disorder	Moderate depressive disorder	Major depressive disorder	Bipolar disorder	Adaptative disorder	Anxiety disorder	Another diagnosis: a detail which one
		Indicate if you have children	Has no children	One child	Two children	Three children	Four children	5 or more children	
		Genre	Woman	Man	Other				
		Age (years)	18–28	28–38	38–48	48–58	58–68	68–78	> 78
		Level of schooling							
			Basic education	Secondary education	Technical education	University education			
		Cohabitation	Alone	With friends	With couple	With family			
		Marital status	Single	Married	Separated	Widow			
		Occupation	With employment	Student	Unemployed	Housewife	Retired		
		Id							
		Name							

The results identified whether the participant being evaluated was in a fragile state that made him or her vulnerable to actively thinking about suicide or attempting to commit suicide. As mentioned above, this assessment tool shows protective and risk factors for each patient, which might guide evaluators and clinicians toward indicating aspects of interest for psychotherapeutic intervention. A patient profile description was elaborated in terms of the following:

- Feelings of satisfaction/dissatisfaction with life.
- State of satisfaction/dissatisfaction with oneself and achievements.
- Reasons to live/to stay alive if one is thinking about attempting suicide.

## Results

The raw results of the analysis, and later results including expert analysis, showed that there were categories that interacted to configure a state of vulnerability. These attributes differentiated the state stems from trait stems. In addition, some of the attributes were factors that had an impact on suicide risk, while others coexisted or appeared as symptoms (or vulnerability). The following interrelationships between attributes were observed: 1) having an impact on vulnerability; 2) coexisting and being relatively stable over time; and 3) being part of the state of psychological vulnerability.

The attributes that had an impact on state stems of vulnerability were sociodemographic in nature, such as gender, age, marital status, number of children or coinhabitants, level of schooling, occupation, and diagnosis. The attributes that coexisted, which were trait stems, were relatively stable and were associated with personality. These attributes were from the quadrant of self-criticism/dependency depressive experience styles, as observed in personal descriptions with regards to the following: feeling aligned with one’s own plans and goals, sometimes feeling empty inside, feeling afraid when alone, feeling good about oneself whether one succeeds or fails, being concerned about relationships with others and what other can provide, and feeling satisfied with oneself and what one has achieved. These attributes also included ascribing more or less importance to reasons for attempting suicide, such as thinking that one can adapt to problems or that only God has the right to end a life; not wanting to die; thinking that life is all that we have and is better than nothing; knowing that feeling bad will not last forever, no matter how bad it becomes; wanting to experience everything that life has to offer; loving oneself enough to stay alive; considering life to be too beautiful and precious to bring to an end; thinking that a solution can be found to any problem; feeling love for life; feeling too stable to kill oneself; hoping that things will get better and that the future will be happier; seeing no reason to hurry death; and considering the idea of suicide as totally incomprehensible. According to their joint probabilities, these variables supported the formation of a state of vulnerability, which were highly changeable and could be modified, such as feeling satisfied/dissatisfied with oneself and with life, being interested in something, having thoughts about ending one’s life, feeling like a happy person, and feeling content with oneself and with life. Details of these variables can be seen in the Psychological Vulnerability Questionnaire shown in Table 7.

The use of this instrument provided specific recommendations for the evaluator, such as in the following example: for one participant, the answers provided placed the participant in the risk of suicidal behavior group with a probability of approximately 60%. In the presence of suicidal ideation, feelings of emptiness and dissatisfaction with oneself should be given special attention, and the expectations the patient has regarding interpersonal relationships and the presence of hopelessness should be evaluated.

## Discussion

The general purpose of our work was to identify more accurately which clinical aspects of mood and anxiety disorders are most likely to be seen in a patient at risk of suicide. This can be considered a clinical study because we sought to establish a tool that recognizes risk based on a select group of variables, which may then be included in psychotherapeutic interventions. We recognize these findings to be part of a broader initiative to explore new methods and work strategies, whose applications and usefulness are just beginning to be known.

In this study, we established conditional dependence relationships between factors (transitory or stable) that were predominant in patients with suicidal ideation or who had recently attempted suicide. By systematizing variables with the Bayesian network method, we could visualize an image that showed directed graphs with the nodes and edges representing random variables in conditional dependency relationships or the correlations among them, which emerged within a time context. The relative values of each variable were defined by the algorithm that could assess whether a patient was or was not in a state of vulnerability. This method of organizing and selecting rich data could assist in recognizing relevant aspects of SB, which could be focused on in early interventions.

Our results could be compared to those yielded by traditional statistical methods with different metrics. However, the purpose of this study was not to make comparisons with other methods. Instead, we aimed to gain greater knowledge on how data mining can be used to understand suicidal behavior [8].

The results from analyzing Bayesian networks are considered to be measures used to adjust training data. Further studies to validate the instrument are necessary, which could allow us to obtain accuracy metrics of its predictive power in terms of precision, specificity, and sensitivity. New evaluations from hospitalized and outpatient participants should be considered for future studies validating the model.

The sequence of data mining analyses (SVM and DT) provided a fine-grained view of the data, which illuminated interrelationships that could configure vulnerability states. The use of the BN technique assisted in identifying individual treatment indications given the following conditional dependence relationships between attributes: having an impact on vulnerability, coexisting and being relatively stable over time, and being part of a state of psychological vulnerability. These conditional dependence relationships can be considered protective and risk factors, depending on the case at hand, and can be related to degrees of satisfaction with oneself, with others, and with life as well as the relative to values assigned to reasons for staying alive.

Our sample included patients with mood and anxiety symptomatology who were willing to participate in this study. We decided to restrict our sample to the most frequent group of patients in clinical practice. With the aim of extending its use to other diagnostic groups, our instrument should be tested on samples with greater diagnostic variety. The results reported here can contribute to better recognition of a mental state that might precede SB. Similar to targeting obesity, high blood pressure, and a sedentary lifestyle, which might decrease the risk of cardiovascular disorders, our study intended to identify factors that could be focused on in early interventions.

The items in the proposed instrument refer to patients’ feelings toward themselves, life in general, and reasons for living, making the instrument suitable for use in a range of contexts aside from mental health institutions, such as educational environments or policing. With the use of this psychological evaluation questionnaire, it was possible to create a tool to assess general states of vulnerability and identify individuals who may be at risk of SB and who need to be directed to a specialist for further clinical assessment.

In addition, this instrument has a significant advantage over specific scales or questionnaires regarding suicidal ideation or behavior since it provides suggestions of areas for the patient to work on during psychotherapy in terms of the patient’s psychologically strong and fragile aspects. Likewise, this model provides an advantage over traditional instruments, as traditional instruments may have adverse effects when used by poorly trained psychologists or psychiatrists (Arensman personal communication September 10th, 2017 NASP). Our work illuminated the use of traditional variables in a different way. As international research has suggested, this different approach could be considered the start of a new paradigm. Our overall aim was to contribute to public health with a new tool that aims at recognizing specific profiles of patients who might be in states of vulnerability. Interventions based on this tool could be adapted to specific characteristics found in each patient. This is a model that might be welcomed by those who support personalized interventions.

The process of evaluation and first intervention with this type of tool should broaden patients’ perspectives by allowing them to explain the subjective logic behind their suicidal behavior and identify what affects them and what helps them in times of suicidal crisis. This type of tool could enable interventions to focus upon configurations of particular factors that can be enhanced or diminished in psychotherapy. Psychotherapeutic work could then focus on promoting emerging aspects such as current coping abilities, personal stability, the capacity to regulate emotions, feelings of personal competence, and feelings of satisfaction with oneself and with achievements. Such work could also serve to develop skills and areas of interest in life, to strengthen one’s reasons to stay alive, and to reinforce support networks.

Likewise, this type of tool could allow patients to deepen their psychological elaborations of their feelings of disability, their capacity for emotional regulation and their dissatisfaction with oneself and with life. Furthermore, it may help in identifying and changing patterns of negative beliefs about oneself and others and mitigating traits of extreme dependence or extreme self-demand to alleviate despair and hopelessness.

Limitations of our findings should not be disregarded. As mentioned previously, our sample only included patients with major depression using DSM IV criteria. The diagnostic criteria for depression and anxiety in the DSM V contain modifications that we will have to include in the application of the results of our work.

Another limitation of this study is the exclusion of patients who had addictions, eating disorders, psychotic disorders or cognitive disorders. This decision was methodological, with the purpose of controlling the diagnostic variable in the detection of psychological vulnerability. It would be advisable for future research to include these pathologies in the study of psychological vulnerability to suicide.

The absence of data from those who decided not to participate and from those who, having initially accepted, later withdrew from the study, is another limitation of these findings.

Age needs to be examined more closely in further studies since our sample included patients above 14, and responses were similar across different age groups. Regarding the relationship between income and suicidal behavior, although there is evidence of an association between the two variables, this association needs to be explored further ( [20]; Knipe et al., 2015 [21];). The present study did not reveal differences relative to income and suicidal behavior since the sample included a relatively heterogeneous level of income. Finally, information that was not provided by individuals who chose not to participate in the study may have changed the results if it had been included.

## Conclusion

As a recent review of the use of DM and AI to study SB showed, our work is consistent with the experience of many authors who supported the use of these techniques to study SB [8]. However, our interest in more precisely identifying the major factors associated with the mental state that precedes SB and their configuration is a new approach. This is an important methodological difference from the work cited in the mentioned review, as we have discussed in this manuscript. The usefulness of this new approach will need to be evaluated in subsequent studies.

While it is not possible to predict suicide risk today, we can more accurately explore the state of mind that patients experience closest to the time of suicidal behavior". The usefulness of our results must be evaluated prospectively. To do so, we must follow the evolution of behavior based on the variables that show these results. We are already doing this in a sample of emergency services patients. We are currently following a sample of patients using several clinical instruments to evaluate the usefulness of our tool.

Our findings allow for the proposal of new goals for further studies. First, we need to evaluate the usefulness of this model in samples with broader diagnostic profiles (since these findings are applicable only to patients with mood disorders). We also believe that it is convenient to classify risk groups according to age and/or pathology to study whether the risk profile of SB has differences in these groups with respect to others. All of these steps are taken with the purpose of recognizing risk patterns that can be highly specific and personal.

## Data Availability

The dataset of this research is deposited in a publicly available repository in an Excel spreadsheet. Synapse ID: syn20048270 DOI:10.7303/syn20048270.1. Conditions for use: Controlled Use.

## Footnotes

1 Conditional dependency relationships: measures of the probability of an event occurring given that another event has (by assumption, presumption, assertion or evidence) occurred [14].

2 Support vector machine models: Supervised learning models associated with learning algorithms that analyze data used for regression and classification analysis. Starting with a set of training examples, each marked as belonging to one or the other of two categories, an SVM training algorithm builds a model that assigns new examples to one category or the other, hence resolving a non-probabilistic binary linear classifier [6].

3 Decision tree technique: A model of computation in which an algorithm is considered to be a sequence of branching operations based on comparisons of some quantities, the comparisons being assigned the unit computational cost. The branching operations are called “queries or tests”. The algorithm may be considered a computation of a Boolean function where the input is a series of queries and the output is a final decision, in which every query is dependent on previous queries or tests [7].
